# In Web We Trust: The Promised Cannabidiol Effects on Obesity as a Matter of Language and Marketing on Webpages

**DOI:** 10.2174/011570159X333465241121184404

**Published:** 2025-01-02

**Authors:** Roberta Roberti, Carla Comacchio, Marco Colizzi, Carlo Augusto Mallio, Emilio Russo, Gianfranco Di Gennaro

**Affiliations:** 1 Department of Health Sciences, University of Catanzaro “Magna Græcia”, Viale Europa, 88100 Catanzaro, Italy;; 2 Unit of Psychiatry and Eating Disorders, Department of Medicine (DMED), University of Udine, 33100 Udine, Italy;; 3 Fondazione Policlinico Universitario Campus Bio-Medico, Rome, Italy;; 4 Research Unit of Radiology, Department of Medicine and Surgery, Università Campus Bio-Medico di Roma, Rome, Italy

**Keywords:** Cannabidiol, weight loss, world wide web, health-related information, obesity-related terms, online information, non-English-speaking countries

## Abstract

**Background:**

Today more and more people search the web for health-related information, risking to come across misinformation and biased content that may affect their treatment decisions. Cannabidiol (CBD) is among the products for which beneficial effects have been claimed, often at the expense of the risks; further keeping in mind unreliable information reported on products themselves.

**Objective:**

This study evaluated the quality of information retrieved by Google on the potential effects of CBD on weight management, also comparing Italian and English contents, hypothesizing generally low quality and language-driven differences in offered information.

**Methods:**

Queries regarding cannabidiol and obesity-related terms were entered into Google, ranking the first 50 webpages from both merged Italian and English results for analysis.

**Results:**

Of the outputs, 37 Italian and 27 English websites addressed the topic and were not related to medical literature. As expected, a substantial proportion of information was of low quality, with English sites performing better (29.6%) than Italian ones (54%, *p =* 0.052) in terms of “JAMA benchmarks” for trustworthiness of information. Also, while most English sites were “Health portals” (40.7%) with neutral stance toward CBD (74.1%), Italian ones were predominantly “commercial” (78.4%, *p* = 0.001) and promoting CBD use (89.2%, *p* < 0.001).

**Conclusion:**

Findings suggest the need for better online information, especially in non-English-speaking countries, as scarce and unequal information can lead people to make poor health choices, with potentially harmful consequences.

## 1 INTRODUCTION

In recent years, the global prevalence of obesity has risen, constituting a pressing public health concern [1]. As traditional interventions struggle to address the multifaceted nature of obesity, novel approaches have garnered attention [2-4]. Among these, cannabidiol (CBD), a non-euphoric constituent of Cannabis sativa, has emerged as a promising candidate due to its supposed therapeutic properties, including its potential to modulate metabolic processes [5-7]. As interest in CBD’s role in weight management grows, individuals turn to the vast expanse of the internet for information, as much as for many other potential medical information and self-medication or personal use. However, among the abundance of online resources, the reliability and quality of information concerning any specifical medical communication, also including CBD’s effects on obesity, remain uncertain [8, 9].

The internet has become an increasingly popular source for health information, with a large percentage of subjects admitting that the decisions about how to treat an illness or condition have been influenced by search results [10]. The easy access to this ubiquitous platform for disseminating information, comes with inherent challenges, notably the proliferation of misinformation and biased content [11]. Considering the mainly enthusiastic interest surrounding CBD and cannabis derived products in the last decades as much as its economic value, individuals navigating the digital realm encounter a plethora of websites asserting various claims regarding its efficacy in mitigating obesity-related concerns. However, it is not easy for not experts to determine the quality of the contents, and the absence of standardized guidelines and regulatory oversight renders discerning the credibility of such information a formidable task.

Moreover, the dynamic nature of the internet perpetuates the dissemination of anecdotal evidence, speculative claims, and commercial agendas, often overshadowing scientifically validated insights [12]. Consequently, individuals seeking reliable information may be susceptible to misconceptions, potentially hindering informed decision-making regarding CBD’s role in managing obesity and weight loss. Nevertheless, there are relevant risks in the use of CBD-based products linked to a lack of reliability regarding what is reported on the label and the real content of accessible products as much as their claimed effects [9, 13]. Consumers and practitioners should remain cautious of unregulated and often-mislabeled CBD products due to the risks of taking too much CBD (*e.g*., drug-drug interactions, liver enzyme elevations, increased side effects) and the consequences of taking too little (*e.g*., no clinical benefits due to underdosing) [14].

Finally, it is well known that for several medical topics, the quality of online information can vary depending on the country and language used by the internet user to conduct the search. More precisely, American and English websites, but more generally Western websites, tend to exhibit higher quality compared to websites from other regions of the world [15-17]. Understanding the critical importance of evidence-based information in healthcare decision-making, it becomes imperative to assess the quality and reliability of online resources pertaining to CBD and obesity.

This study was aimed to evaluate the quality of information retrieved by Google on the potential effects of CBD in the context of obesity management and weight loss. Our hypothesis was that the quality of information reported on the websites would be very poor and linked to marketing. Further, as literature suggested country-related differences in the level of web information and these may have an influence on consumers’ perceptions and attitudes, we searched for results in Italian and in English to compare the quality of contents.

## 2 METHODS

The query “Cannabidiol and Obesity”, “Cannabidiol and Overweight”, “Cannabidiol and Weight Management” and the Italian counterparts “Cannabidiolo e Obesità”, “Cannabidiolo e Sovrappeso” and “Cannabidiolo e Gestione del Peso”) were entered into Google on March 15^th^, 2024, by using “.co.uk” and “.it” country code domains respectively. The first 50 organic search results from each query of both British and Italian search engine results pages (SERPs) were merged and duplicated results were removed. The first 50 ranked webpages from both merged Italian and English results were analysed.

We refrained from using queries with highly technical or scientific language as they do not reflect the typical online behaviour of the average internet user and tend to inflate the perceived quality of information, as indicated in the literature [18, 19].

To counteract the potential impact of the “filter bubble effect”, where search results are personalized based on individual preferences, we conducted Google searches in a “private mode” window using the Google Chrome browser. Prior to each search, we cleared the browsing history and cache, and employed a dedicated Google account that had never been used before for this purpose.

The trustworthiness of all downloaded websites was assessed by the so called “JAMA benchmarks” tool, a 4-points rating scale that assesses whether the following distinct criteria are met: “authorship” (*Authors and contributors, their affiliations, and relevant credentials should be provided*), “attribution” (*References and sources for all content should be listed clearly, and all relevant copyright information noted*), “disclosure” (*Web site ownership should be prominently and fully disclosed, as should any sponsorship, advertising, underwriting, commercial funding arrangements or support, or potential conflicts of interest*) and “currency” (*Dates that content was posted and updated should be indicated*) [20].

To assess the completeness of information regarding the use of CBD in managing obesity and overweight, we investigated whether websites provided information on expected effects of CBD, side effects, side effects due to the route of administration, interactions with other drugs, dosage, at-risk populations, contraindications, storage, administration methods, and mechanism of action. Finally, for each website, we evaluated its stance (“promoting”, “neutral”, “demonizing”) towards CBD and classified the website based on its type (“journalistic”, “professional”, “governmental”, “non-profit”, “commercial”, and “health portal”).

Two researchers (ER, MC) independently analysed the websites. In cases of disagreement, a third author (GDG) arbitrated. According to the literature, we adhered to the “three-click rule” for information gathering, meaning we explored each webpage within three clicks [21].

### 2.1 Statistical Analysis

The data were summarized using counts and percentages as it comprised solely categorical variables. We removed websites linking to medical literature articles from the analysis to eliminate a language bias, as these websites were exclusively present in the English SERP.

A “JAMA score” was computed by assigning one point to each of the four JAMA criteria when they were met. Webpages that achieved a score of 3 or 4 were categorized as informative “high-quality” webpages, as recommended in the literature [22, 23]. The distribution of the evaluated criteria between the English and Italian SERPs was analysed using Pearson’s Chi-Square test. In cases of low sized (< 5) cells, Fisher’s exact test was employed.

## 3 RESULTS

Of the downloaded websites, 37 Italian and 27 English websites respectively addressed the topic of CBD and overweight/obesity and were not related to medical literature. As reported in Table **1**, 70.4% of English websites could be classified as “high quality” compared with 46% of Italian websites, with a difference in proportions approaching statistical significance (*p =* 0.052). Regarding the completeness of information, English websites provided significantly more frequent information compared with Italian websites on the following criteria: side effects (66.7% *vs.* 8.1%; *p <* 0.001), side effects due to the route of administration (44.4% *vs.* 2.7%; *p <* 0.001), interactions with other drugs (74.1% *vs.* 13.5%; *p <* 0.001), at-risk populations (44.4% *vs.* 10.8%; *p =* 0.003), and contraindications (37.0% *vs.* 2.7%; *p <* 0.001). No websites demonizing the use of CBD for managing overweight and obesity were identified. Specifically, Italian websites (89.2%) were statistically (*p <* 0.001) more frequently promoting its use compared to English websites (25.9%). Importantly, it is worth noting the difference (*p =* 0.001) in the types of websites being presented, with Italian websites predominantly being “commercial” (78.4%) while English users were mainly exposed to “Health Portals” (40.7%).

## 4 DISCUSSION

In this study, we evaluated and compared the quality of information provided by the first 50 websites returned by Italian and English SERPs (37 Italian and 27 English) on the potential role of CBD in managing obesity and overweight.

In recent years, CBD has gained significant popularity for its potential therapeutic properties; two specific products are currently approved as a drug (*i.e.,* tetrahydrocannabinol and cannabidiol in a 1:1 molecular ratio and a plant-derived, highly purified CBD formulation for the treatment of multiple sclerosis-related spasticity and some specific epileptic syndromes, respectively) but many other applications seem to be promising, mainly but not exclusively in the neurologic field [9, 24, 25]. Unfortunately, the growing interest in CBD treatments has exceeded the scientific research and regulatory advancement, resulting in a confusing scenario of misinformation and unsubstantiated health claims [9, 26]. The lack of strict regulation in the CBD market has led to the availability of not standardized or well-studied products containing CBD, easy to find also in convenience stores and online. Together with a promised but undemonstrated efficacy, these commercialized CBD preparations can be contaminated or have a wide variability in CBD concentrations, increasing the users’ risk of undesirable effects [13, 24, 27]. Therefore, getting correct information becomes of paramount importance, even more in a research field in which results are still preliminary, like this study topic. As an example, there are reports of intoxications due to contamination of cannabidiol oils, therefore, this is not only a matter of not respecting the label and the potential use but also a safety warning [13, 27, 28].

As expected, we found some differences in the trustworthiness of the websites and in the completeness of information retrieved by searching in two different languages. The proportion of websites classifiable as of “*high quality*” according to JAMA score was higher when searches were conducted in English; the JAMA score most frequently reported was 2 for Italian websites and 4 for those English. The major contributor to this difference seems to be attributed to Authorship, since English websites provided more frequently than those Italian information on Authors and contributors. No difference exists regarding disclosures, fully discussed by both. All the English websites provided information on expected effects of CBD, but approximately half of them informed about side effects. Therefore, albeit there was a statistically significant difference between Italian and English websites in providing information on CBD safety, this data is however lacking despite its importance. The adverse events associated with CBD as a drug are well-known (somnolence, diarrhoea, decreased appetite), and commonly mild [29], but are managed by physicians with expertise in the field. In a context of such scarce information, self-medicated subjects may be completely unaware of being at risk of CBD-related side effects. Additionally, little information exists on the population at risk and warning, which was provided more frequently when searches were conducted in English, but still in less than half of the analysed websites. It is worth noticing the very low percentage of Italian websites which warn readers about drug interactions associated with CBD (13.5% *vs.* 74.1%). This finding raises further safety concerns. Cannabidiol can interact with the metabolism of many drugs, and people taking medication for chronic conditions may also be exposed to harms and side effects due to CBD-mediated drug-drug interactions [24]. Most Italian and English websites did not provide information about CBD dose and storage, about half of them describe how CBD is administered, almost all reported CBD mechanism of action. However, especially about this latter data, it is important to highlight that, even if available, the content of the information is generally incorrect often not even referring to mechanisms potentially involved in obesity or weight loss.

Summarizing, our hypothesis that the quality of information reported on the role of CBD in managing obesity/ overweight is poor and differs between countries seems to be verified. On the other hand, the link between information and marketing was shown only by Italian websites, which exposed users mainly to commercial contents. Not surprisingly, these webpages had a promoting stance towards CBD. The high number of commercial websites retrieved by Google might be proportional to the high total number available on the Internet. This is partly true, since a study authored by Aslam and colleagues [30] showed that the majority of results obtained by searching for information on antioxidant were commercial and news websites, but despite this, they were ranked low. Indeed, the first ten websites returned were mainly health portals, government, and professional websites. As the ranking showed the same trend as the JAMA score (the higher the score, the higher the ranking), it has been hypothesized that the intrinsic dimensions of the quality of information might have a role in the ranking algorithm [30]. Due to the small number of analysed webpages, we could not perform a multivariate analysis to assess whether the quality of the information was associated with the type of website. However, English results, which had a higher JAMA score, were mainly constituted by “Health Portals” with a neutral stance towards CBD, and ranked low commercial websites. These findings are consistent with literature data [30-32], contrasting our hypothesis that Google results would have favoured marketed-oriented and promotional sources. Website visibility plays a crucial role, given that, in most cases, users do not go beyond the first results in the SERP [33]. The low ranking of commercial websites might also be the result of an adaption to users’ behaviour, as the perception of commercial intentions has been found to negatively influence trust in online health advice [34]. A possible explanation of the higher number of commercial websites observed in the Italian SERPs might be linked to the linguistic register. We expected to exclude from the analysis a higher number of English websites as they can directly link to medical literature articles, and this happened notwithstanding the highly technical or scientific language was avoided in selecting queries. We can speculate that, compared with English users, Italians had to select a more formal and technical language to find information of high quality, leading to access inequalities. To confirm this hypothesis, we would have to assess the websites returned using other queries, since search results change according to the terms used. These differences also represent a limitation to the generalizability of our findings, as well as the lack of standardized criteria to assess the completeness of information, which have been established from a strictly medical perspective. Different healthcare professionals, or users, might have been interested in knowing additional information not included among the selected criteria.

## CONCLUSION

In conclusion, the quality of the information provided by Google to individuals exploring CBD potential in managing obesity and overweight seemed to be higher in English than in Italian websites, but overall scarce. Searching for results in Italian, users were mainly exposed to commercial websites promoting CBD, whereas “Health portals” with neutral stance toward CBD were more commonly retrieved by Google using English queries. This difference might be due to the direct link of the English language to medical literature, even when technical terms were avoided. Further studies on the impact of linguistic register are needed to clarify whether Italians had to use a more formal language to access high-quality information. As results change according to the queries and no-standardized criteria were available to evaluate the completeness of information, we must be careful in generalizing our results. However, in such a preliminary research field, it is important to underline the need for better information: scarce and unequal information can lead people to make poor health choices, with potentially harmful consequences.

## Figures and Tables

**Table 1:** Characteristics of the analysed web pages, stratified by SERP (English and Italian). Between-SERPs differences in frequency distributions assessed by Pearson’s Chi-Square test or Fisher’s exact test when cell size < 5.

-	**-**	**Overall (n=64)**		**Italy (n=37)**		**UK (n=27)**		
		**n**	**%**	**n**	**%**	**n**	**%**	***p***
Authorship	Yes	35	54.7	16	43.2	19	70.4	0.031
	No	29	45.3	21	56.8	8	29.6	
Attribution	Yes	39	60.9	19	51.4	20	74.1	0.066
	No	25	39.1	18	48.6	7	25.9	
Disclosure	Yes	60	93.8	35	94.6	25	92.6	1.000
	No	4	6.2	2	5.4	2	7.4	
Currency	Yes	47	73.4	25	67.6	22	81.5	0.213
	No	17	26.6	12	32.4	5	18.5	
JAMA	0	2	3.1	1	2.7	1	3.7	0.026
	1	7	10.9	5	13.5	2	7.4	
	2	19	29.7	14	37.8	5	18.5	
	3	8	12.5	6	16.2	2	7.4	
	4	28	43.8	11	29.7	17	63	
High quality (JAMA>2)	Yes	36	56.2	17	46	19	70.4	0.052
	No	28	43.8	20	54	8	29.6	
Expected effects	Yes	49	76.6	22	59.5	27	100	<0.001
	No	15	23.4	15	40.5	0	0.0	
Side effects	Yes	21	32.8	3	8.1	18	66.7	<0.001
	No	43	67.2	34	91.9	9	33.3	
Side effects routes	Yes	13	20.3	1	2.7	12	44.4	<0.001
	No	51	79.7	36	97.3	15	55.6	
Interactions	Yes	25	39.1	5	13.5	20	74.1	<0.001
	No	39	60.9	32	86.5	7	25.9	
Dosage	Yes	28	43.8	14	37.8	14	51.9	0.264
	No	36	56.2	23	62.2	13	48.2	
Pop at risk	Yes	16	25.0	4	10.8	12	44.4	0.003
	No	48	75.0	33	89.2	15	55.6	
Warnings	Yes	11	17.2	1	2.7	10	37.0	<0.001
	No	53	82.8	36	97.3	17	63.0	
Storage	Yes	12	18.8	10	27.0	2	7.4	0.058
	No	52	81.2	27	73.0	25	92.6	
Administration	Yes	45	70.3	25	67.6	20	74.1	0.574
	No	19	29.7	12	32.4	7	25.9	
Mechanism of action	Yes	57	89.1	34	91.9	23	85.2	0.443
	No	7	10.9	3	8.1	4	14.8	
Stance on CBD	Demonizing	0	0.0	0	0.0	0	0.0	<0.001
	Neutral	24	37.5	4	10.8	20	74.1	
	Promoting	40	62.5	33	89.2	7	25.9	
Type	Commercial	38	59.4	29	78.4	9	33.4	0.001
	Journalistic	5	7.8	1	2.7	4	14.8	
	No-Profit	1	1.6	1	2.7	0	0.0	
	Health Portal	14	21.9	3	8.1	11	40.7	
	Professional	6	9.4	3	8.1	3	11.1	

## Data Availability

All data generated or analysed during this study are included in this published article.

## LIST OF ABBREVIATIONS

CBD: Cannabidiol
SERPs: Search Engine Results Pages
